# Three-Dimensional Continuous Displacement Measurement with Temporal Speckle Pattern Interferometry

**DOI:** 10.3390/s16122020

**Published:** 2016-11-29

**Authors:** Jie Qin, Zhan Gao, Xu Wang, Shanwei Yang

**Affiliations:** Key Laboratory of Luminescence and Optical Information of Ministry of Education, Beijing Jiaotong University, Beijing 100044, China; 14121619@bjtu.edu.cn (J.Q.); 15121631@bjtu.edu.cn (X.W.); 15121635@bjtu.edu.cn (S.Y.)

**Keywords:** displacement sensor, three-dimensional measurement, temporal spackle pattern interferometry, wavelet transform

## Abstract

A speckle interferometer which can measure whole field three-dimensional displacements continuously and dynamically has been built. Three different wavelength lasers are used to produce the speckle interferograms of the two in-plane displacements (displacements in the *x*- and *y*-direction) and one out-of-plane displacement (displacement in the *z*-direction), respectively. One color CCD camera is employed to collect these mixed speckle interferograms simultaneously. The mixed interferograms are separated by the Red, Green and Blue channels of the color CCD camera, and then are processed by the wavelet transform technique to extract the phase information of the measured object. The preliminary experiment is carried out to demonstrate the performance of this new device.

## 1. Introduction

Three-dimensional (3D) displacement, which can be translated further into 3D strain and stress, is the key parameter for design, manufacturing and quality control [1]. Due to rapid development of the manufacturing industry, especially for automobile and aerospace applications, the support of dynamic and high-accuracy 3D displacement measurement is required.

Flynn used five object beams and three colors to measure 3D deformations [2]. Each color was separated through dichroic filtering before being recorded by a separate CCD camera. The Fourier-transform method was used to achieve high displacement sensitivity. However, the application of three CCD cameras means that the calibration [3] and algorithm are complex, and the measuring accuracy is affected. Therefore, a single color CCD camera is used to avoid these problems.

Siegmann used a single color camera and a color fringe projector to obtain the real-time 3D displacement maps for the deformable objects. They used a two-dimensional digital image correlation (DIC) to obtain the displacements in the *x*- and *y-*direction and fringe projection to obtain the displacement in the *z*-direction. However, the algorithm for DIC and template matching is complex [4,5,6,7]. Usually the measured resolution of DIC is more than 1 µm [8]. It is lower than the interferometry method, which is less than half a wavelength.

Yano used a color video camera with three CCDs (red, green and blue) to capture the three isolated speckle images generated by three visible semiconductor lasers with adequately-separated wavelengths [9,10]. However, the method is still essentially 3D DIC and cannot realize real-time measurement due to the complex algorithm.

Gao used the non-cube beam-splitter (NCBS) and the 3D optical setup with a fringe carrier method to determine the three phase components effectively [11]. However, like traditional electronic speckle pattern interferometry (ESPI), temporal and continuous deformation cannot be measured. To achieve dynamic continuous measurement, temporal speckle pattern interferometry (TSPI) was proposed by Joenathan, who obtained the phase information by the Fourier Transformation (FT) method instead of the phase-shifting devices [12].

In this paper, such a TSPI-based system has been built. However, different from typical TSPI for 3D measurement, only one color CCD camera is adopted to collect the mixed speckle interferograms, which simplifies the whole system greatly. Moreover, the wavelet transform, which is believed to be more suitable to process the non-stationary signal than the Fourier transform [13], is used to extract the 3D dynamic displacement information of the object. This device, with a simplified configuration, can measure 3D displacements continuously and dynamically. To the best of our knowledge, there is no such system being presented yet.

## 2. Theory

### 2.1. Principle of Out-of-Plane Measurement

**Figure 1:**
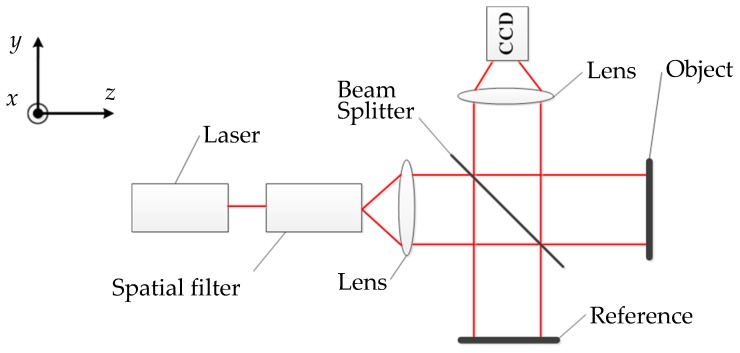
Out-of-plane measurement.

As shown in Figure 1, the configuration is similar to that of the Michelson interferometer, except that two mirrors are replaced with the rough metal plates [14]. The beam emitted from the laser is expanded by the spatial filter, then gets partially reflected and partially transmitted by the beam splitter. One segment travels to the object and the other travels to the reference. The two beams reflected by the object and the reference are made to interfere. The lens is used to focus the speckle interferograms onto the sensor of the color CCD camera. A series of frames of the speckle interferograms are recorded as the object is being displaced. Each frame is then a record of the speckle interferograms and their intensity at that instant of time. These interferograms are processed by the wavelet transform and the phase unwrapping algorithm to acquire the phase information. Finally, the object displacement is obtained.

Before the displacement of the object, the interference intensity of one point on the object can be expressed as:

(1) $$I\left(x,y,t\right)=I_{0}\left(1+Vcos\left[\Phi_{0}\left(x,y\right)\right]\right)$$

where $I_{0}$ is the average intensity of the interference field. *V* is the modulation visibility. $\Phi_{0}\left(x,y\right)$ is the initial phase.

After the motion of the object, the intensity function becomes:

(2) $$I\left(x,y,t\right)=I_{0}\left(1+Vcos\left[\Phi_{0}\left(x,y\right)+\Delta \varphi \left(x,y,t\right)\right]\right)$$

(3) Δφ(x,y,t)=4πΔz(x,y,t)/λ

where Δφ(x,y,t) is the change of the phase, and Δz(x,y,t) is the function of the object displacement in the *z*-direction. 

### 2.2. Principle of In-Plane Measurement

**Figure 2:**
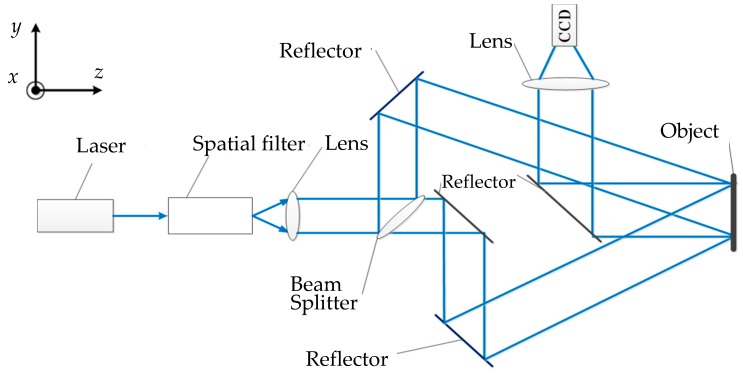
In-plane measurement.

The principle of the in-plane measurement is shown in Figure 2. The beam emitted from the laser is expanded by the spatial filter. Then the beam splitter splits it into two beams. Both of them illuminate on the object surface at the same angle. The scattered back beams from the object form a speckle interferogram and are captured by the color CCD camera.

When the object moves in the *x*- or *z*-direction, the change of the two beams’ optical path remains the same. Thus, the speckle interferogram remains unchanged. If the object moves a small distance in the *y*-direction, the optical path of one beam increases Δy(x,z,t)sin i, and the other one reduces Δy(x,z,t)sin i [15]. Thus, we have:

(4) $$\Delta \varphi =\frac{4\pi }{\lambda }\Delta y\left(x,z,t\right)\text{sin }i$$

Hence, after the motion of the object, the intensity function of the speckle interferogram is:

(5) $$I\left(x,y,t\right)=I_{0}\left(x,y\right)\left\{1+V\text{cos}\left[\Phi_{0}\left(x,y\right)+4\pi \Delta y\left(x,z,t\right)\text{sin }i/\lambda \right]\right\}$$

The intensity of the interferograms will change if the object moves in the *y*-direction. By processing these interferograms with the wavelet transform and the phase unwrapping algorithm, the object displacement can be obtained.

The principle of the measurement in the *x*-direction is as the same as the above method.

### 2.3. Principle of 3D Measurement

**Figure 3:**
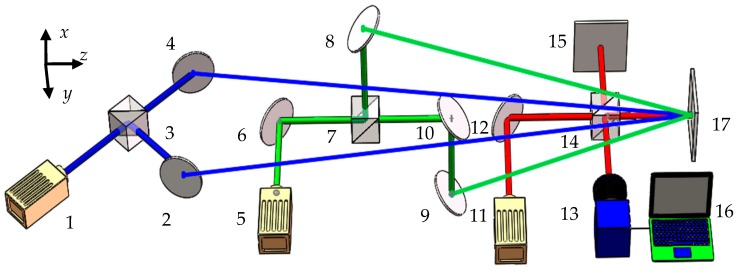
Principle of three-dimensional measurement: 1, 5, and 11 are lasers; 2, 4, 6, 8–10, and 12 are reflectors; 3, 7, and 14 are beam splitters; 13 is the color CCD camera; 15 is reference; 16 is computer; 17 is object.

Based on the principle of the in-plane and the out-of-plane measurement, the three-dimensional measurement system is developed. As shown in Figure 3, the three visible-wavelength lasers are used for the three direction measurements. The blue and green lasers are used to measure the *x*- and *y*-direction displacements, respectively. The red laser is used to measure the *z*-direction displacement. The color CCD camera is used to record the whole process of the object movements.

**Figure 4:**
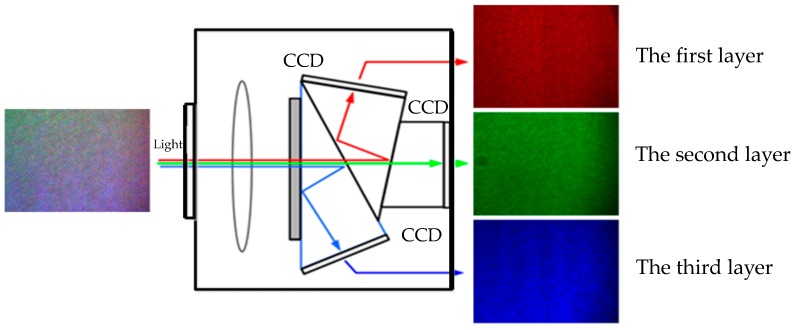
The principle of light separation.

As shown in Figure 4, the mixed speckle interferograms formed by the three lasers are captured by the color CCD camera with three CCDs. The prisms separate the red, green and blue light, then these interferograms are recorded by the corresponding CCD, and each frame of the color CCD camera is divided into three layers to store the data of the R, G and B channels respectively.

In our built system, the data stored in the first, the second, and the third layer correspond to the displacement information of the *z*-, *y*-, and *x*-direction, respectively. Hence, the matrix with the three layers records entire displacements of the three directions. The sampled speckle interferograms are processed by the wavelet transform and the phase unwrapping algorithm to access the phase information. Finally the dynamic object displacements are obtained.

### 2.4. Principle of the Wavelet Transform Method and Signal Processing

The wavelet transform, a tool that excels at multi-resolution and localization in the time- or space-frequency domain, has enjoyed tremendous popularity and notable development during the past decades. The continuous wavelet transform (CWT) [16,17] is defined as:

(6) $$W_{f}\left(a,b\right)=\langle f\left(t\right),\psi_{a,b}\left(t\right)\rangle =\frac{1}{\sqrt{a}}\int_{-\infty }^{\infty }f\left(t\right)\psi^{*}\left(\frac{t-b}{a}\right)dt$$

where a is the scale parameter, b is the shift parameter, *f(t)* is the signal to be analyzed, ψ(t) is the mother wavelet, and $\psi^{*}\left(\frac{t-b}{a}\right)$ is the conjugate function. 

The CWT uses inner products to measure the similarity between a signal and an analyzing function. The amplitude of $W_{f}\left(a,b\right)$ is positively correlated with the similarity of the mother wavelet and the signal. By continuously varying the values of the scale parameter, *a*, and the position parameter, *b*, we can obtain the CWT coefficients $W_{f}\left(a,b\right)$. In this paper, ‘cgau8’ is chosen as the mother wavelet. The wavelet ridge is extracted from the maximum of the coefficients $W_{f}\left(a,b\right)$ of the CWT.

The amplitudes and phases can be calculated by the following equations:

(7) $$A\left(a,b\right)=\sqrt{{\left\{{\left\{I_{m}\left[W_{f}\left(a,b\right)\right]\right\}}^{2}\right\}}^{2}+{\left\{R_{e}\left[W_{f}\left(a,b\right)\right]\right\}}^{2}}$$

(8) $$\varphi \left(a,b\right)=arctan\left\{I_{m}\left[W_{f}\left(a,b\right)\right]/R_{e}\left[W_{f}\left(a,b\right)\right]\right\}$$

where $I_{m}\left[W_{f}\left(a,b\right)\right]$ and $R_{e}\left[W_{f}\left(a,b\right)\right]$ are the imaginary and real parts of $W_{f}\left(a,b\right)$, respectively.

**Figure 5:**
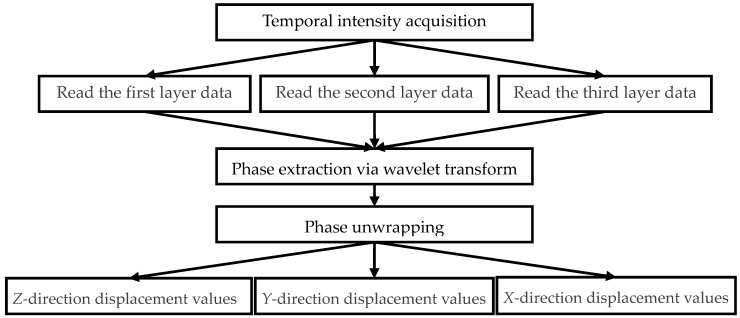
Signal processing flowchart.

After the phase information is computed, the phase unwrapping algorithm is used to obtain the total phase [18]. Finally, according to Equations (3) and (4), the object displacements can be obtained. The whole signal processing flowchart is shown in Figure 5.

## 3. Experiment and Results

### 3.1. Verification Experiment of One Color CCD Camera Light Separation Capability

The wavelengths and powers of the light source are 632.8 nm and 10 mW for the red laser, 532 nm and 50 mW for the green laser, and 473 nm and 50 mW for the blue laser. 

The color CCD camera with 640 × 480 spatial resolution is produced by HITACHI Company (Tokyo, Japan). Compared to a Bayer camera, a one color CCD camera with three CCDs with light separation can obtain more realistic color information.

**Figure 6:**
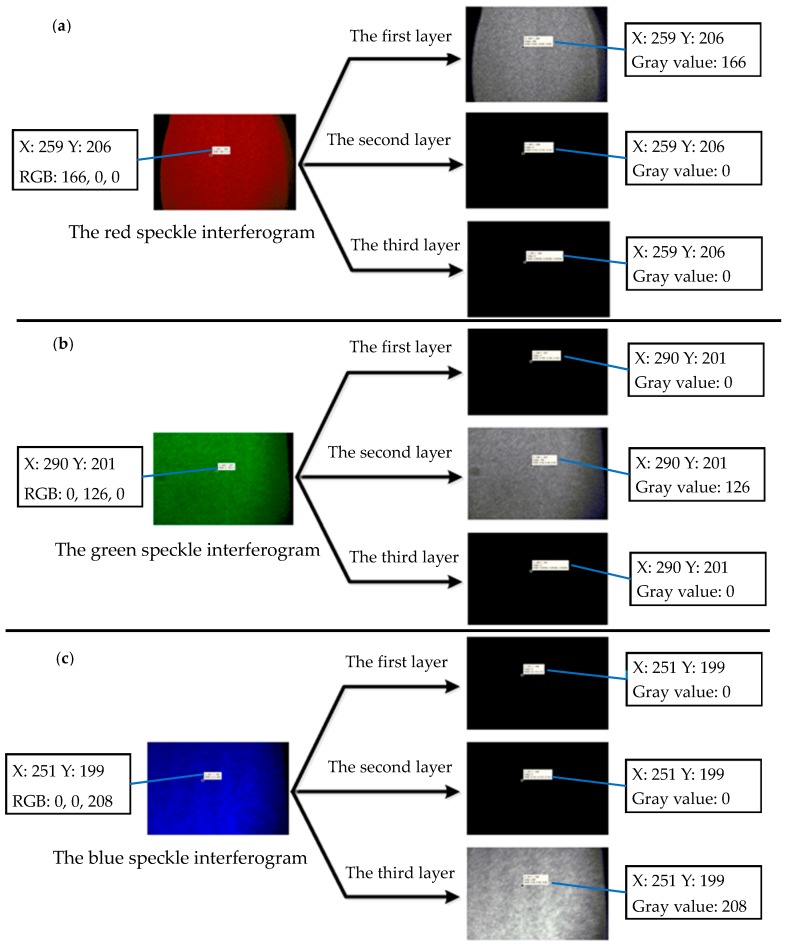
The speckle interferograms and grey-scale maps. (**a**) The red speckle interferogram; (**b**) The green speckle interferogram; (**c**) The blue speckle interferogram.

To test the light separation capability of the color CCD camera, only one laser is allowed to irradiate the object each time, the speckle interferogram of the object is captured by the color CCD camera (these speckle interferograms without subtracting the first frame result in no fringe appearing). As shown in Figure 6, when only the red laser is turned on, the red speckle interferogram of the object and the intensity of one random chosen point on the object are shown on the left-hand side of Figure 6a. The gray-scale maps of the three layers are displayed on the righthand side. Obviously, only the first layer, which is used to record the signal of the R channel, has the gray value signal. The test results of the green and blue lasers are shown in Figure 6b,c, as well. These results prove that the color CCD camera has a very good light separation capability.

### 3.2. The Experiment of the 3D Displacement Measurement

#### 3.2.1. The Experiment

**Figure 7:**
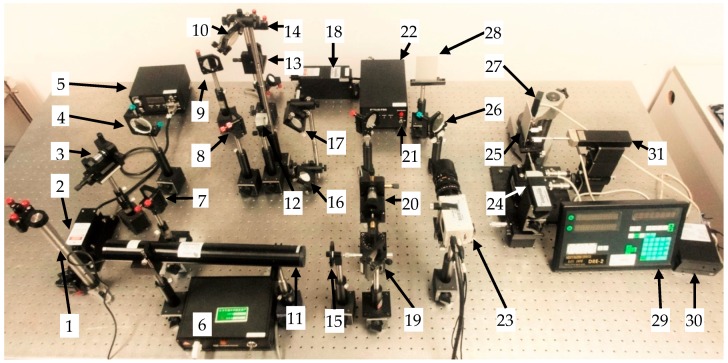
Experiment setup: 1 and 14 are beam lifters; 2, 11, and 18 are the blue, red, and green laser; 3, 13, and 20 are spatial filters; 4, 12, and 26 are beam splitters; 5, 6, and 22 are laser controllers; 7–10, 16, 17, 19, and 21 are reflectors; 15 is an intensity attenuator; 23 is the color CCD camera; 24, 27, and 31 are grating rulers; 25 is the object; 28 is the reference; and 29 and 30 are data displays.

The experiment setup is shown in Figure 7.

The incident angle of the blue laser for measuring the *x*-direction displacement is 18 degrees. The incident angle of the green laser for measuring the *y*-direction displacement is 22 degrees.

**Figure 8:**
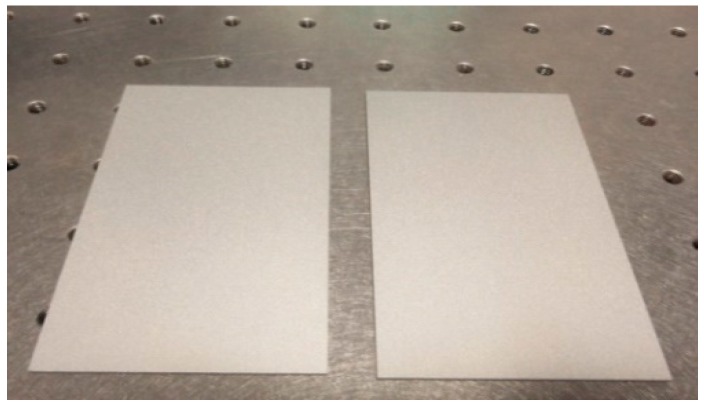
The rigid aluminum plates.

As shown in Figure 8, both of the object and the reference are rigid aluminum plates which have rough surfaces with the same size and material.

**Figure 9:**
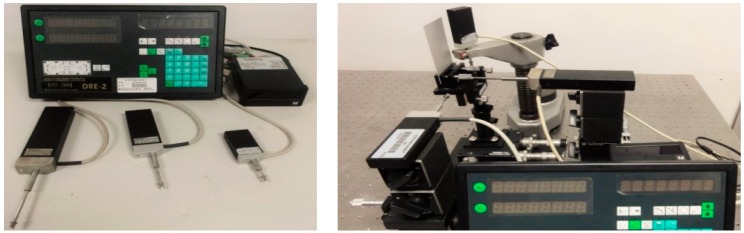
Three grating rulers.

The object is fixed to a THORLABS three-axis flexure stage, with a minimum increment of 20 nm. After careful adjustment, the object surface remains parallel with the *x*-direction so that when the object moves in one direction of the stage manually, the optical path difference in the other two directions will not change. The actual displacement values are measured by the three grating rulers. The accuracy of the grating ruler is 0.1 µm. These grating rulers are shown in Figure 9.

**Figure 10:**
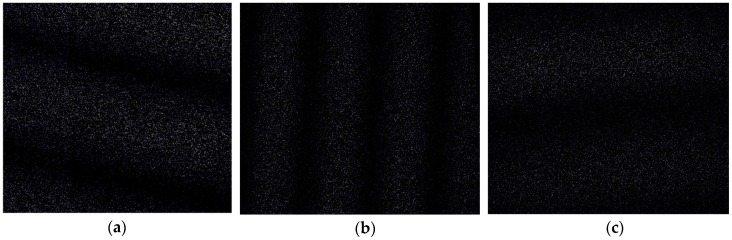
The speckle pattern interferometry fringes: (**a**) the grey-scale map of fringes in the *z*-direction; (**b**) the grey-scale map of fringes in the *y*-direction; and (**c**) the grey-scale map of fringes in the *x*-direction.

As shown in Figure 10, the grey-scale maps of fringes in three directions can be observed by real-time subtraction.

The frame rate of the color CCD camera is 100 frames/s. The whole measuring time is six seconds. Thus, a total of 600 frames are stored. Since the object is a rigid body, an arbitrary point on it is chosen to conduct the measurement.

**Figure 11:**
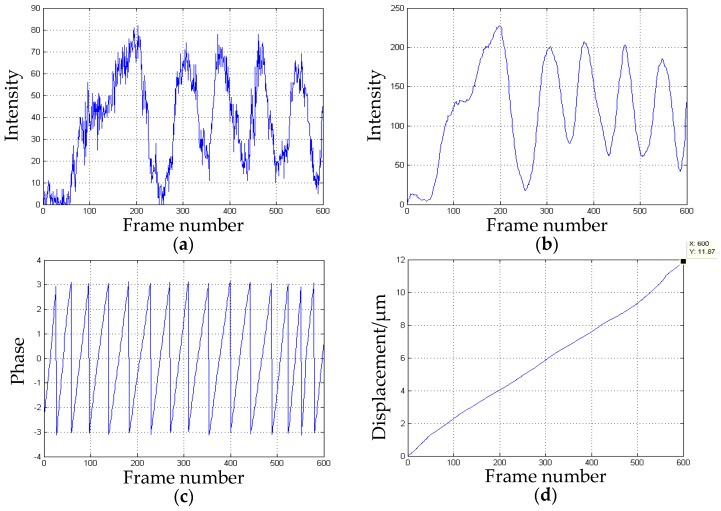
The *x*-direction displacement: (**a**) the intensity distribution over time; (**b**) the intensity after amplification and smoothing processing; (**c**) the discontinuous phase map obtained by CWT; (**d**) the measured displacement over time.

The *x*-direction displacement obtained is shown in Figure 11.

The actual displacement in the *x*-direction measured by the grating ruler is 11.9 µm. The displacement processed by our algorithm is 11.87 µm, therefore, the measured error is 0.03 µm and the relative error is 0.25%.

**Figure 12:**
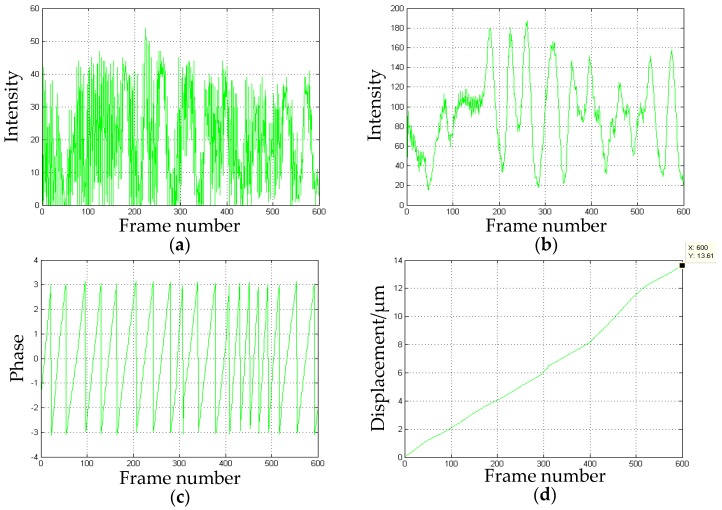
The *y*-direction displacement: (**a**) the intensity distribution over time; (**b**) the intensity after amplification and smoothing processing; (**c**) the discontinuous phase map obtained by CWT; and (**d**) the measured displacement over time.

The *y*-direction displacement obtained is shown in Figure 12.

The actual displacement in the *y*-direction measured by the grating ruler is 13.5 µm. The displacement processed by our algorithm is 13.61 µm, hence, the measured error is 0.11 µm and the relative error is 0.81%.

**Figure 13:**
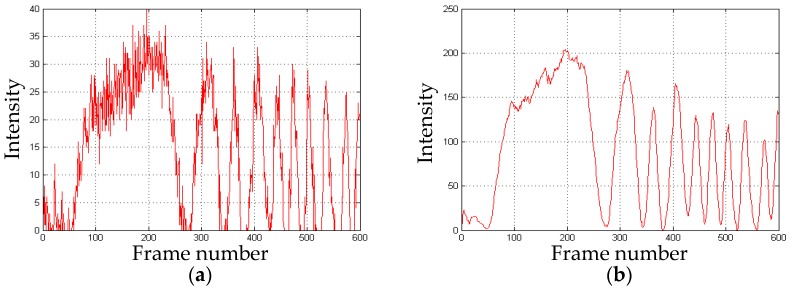

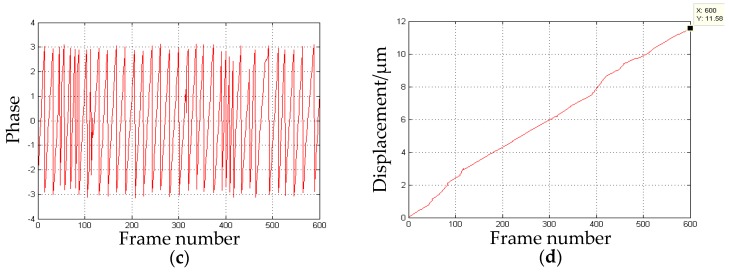
The *z*-direction displacement: (**a**) the intensity distribution over time; (**b**) the intensity after amplification and smoothing processing; (**c**) the discontinuous phase map obtained by CWT; and (**d**) the measured displacement over time.

The *z*-direction displacement obtained by processing is shown in Figure 13.

The actual displacement in the *z*-direction measured by the grating ruler is 11.8 µm. The displacement processed by our algorithm is 11.58 µm, so, the measured error is 0.22 µm and the relative error is 1.86%.

#### 3.2.2. Multi-Detection Points Processing

**Figure 14:**
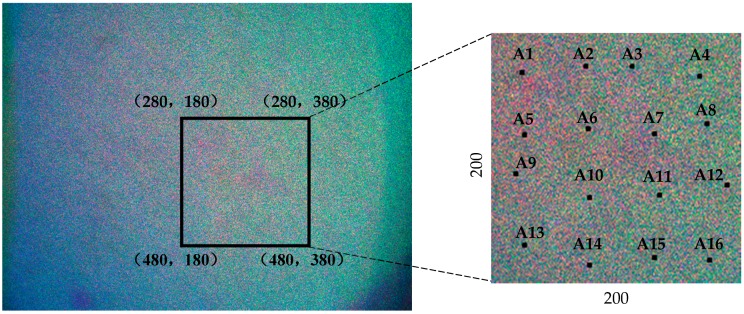
The ROI selected and the points processed.

The system is designed for full-field measurement; therefore, multi-detection points of the object are measured to verify the accuracy of the system. The region of interest (ROI) which is chosen arbitarily, is shown in Figure 14. Sixteen points inside this area are processed by the algorithm we developed. The measured results are shown in Table 1.

The actual displacements in the *x-*, *y-*, and *z-*directions measured by the grating ruler are 11.9 µm, 13.5 µm, and 11.8 µm, respectively. From the table, the maximum measuring differences between two points in the *x-*, *y-*, and *z-*directions are 0.70 µm, 0.72 µm, 0.69 µm. Meanwhile, the measured errors of almost all processed points are less than half a wavelength.

## 4. Discussion

1. As shown in Figure 11b, Figure 12b and Figure 13b, the width of the first cycle is more irregular and wider than the others. This is caused by the object status changing from the stationary to the motion.
2. The error of the displacement in the *x*-direction is the minimum in the three directions. As shown in Figure 11b, the peak to peak values and shapes of the waves after the first one are almost the same. Moreover, the intervals between the waves are also identical. These led to the most uniform change of the phase processed by the wavelet transform. Hence, a better phase unwrapping results are acquired.
3. The accuracy of the displacement in the *z*-direction is more than 1%. Unlike the common-path layout in-plane measurement, the object and the reference is located in a different path, which is easier to be disturbed by the environment. To improve the accuracy, heterodyne speckle pattern interferometry (HSPI) is expected to be employed in future work.
4. The experiment adopts several points of the metal plate to measure the accuracy of the system which are regarded as measurements of the full-field deformation and displacement. The whole object deformation and displacement will be obtained by processing each point of the object with the same algorithm.
5. The wavelet transform is used to analyze the speckle interferograms. Compared with the Fourier transform, it can show more detail to obtain more accurate displacement information.
6. The measurement resolution of the system is less than half a wavelength, which is limited by many factors, such as the stability of the laser source, sampling rate of the CCD camera used, the disturbance of the environment, etc. In our case, the main factor is the phase unwrapping algorithm, particularly the selection of the mother wavelet. The monitored distance of the system from the object can be from a few meters to 10 meters away in the laboratory, mainly depending on the coherence length of the laser source. In theory, as long as the speckle interferograms can be recorded, the measurement can be completed successfully. However, it is difficult to do this outdoors due to the rapid change of background light intensity, vibrations, and air disturbance, which are common problems in interfering instruments. On the other hand, the camera vibrations caused by ground motion affect the measuring accuracy as well.

## 5. Conclusions

A 3D displacement measurement system with TSPI is presented in this paper. It can capture and process speckle interferograms during the movement of the object continuously and dynamically. Compared with typical 3D ESPI, it can realize 3D dynamic continuous measurement and has a simplified configuration because only one CCD camera is employed. Compared with DIC, it has a higher accuracy and simpler algorithm. The results of the preliminary experiment demonstrate the feasibility of our developed device.

## Figures and Tables

**Table 1:** The measured results of sixteen detection points.

		*x*-Direction		*y*-Direction		*z*-Direction	
Name	Coordinate	Value/µm	Error/µm	Value/µm	Error/µm	Value/µm	Error/µm
A1	(24, 30)	11.99	0.09	13.23	0.27	11.99	0.19
A2	(74, 25)	11.92	0.02	13.67	0.17	11.99	0.19
A3	(111, 26)	12.00	0.10	13.26	0.24	11.88	0.08
A4	(166, 33)	11.89	0.01	13.45	0.05	12.11	0.31
A5	(26, 80)	11.78	0.12	13.21	0.29	11.68	0.12
A6	(76, 75)	11.60	0.30	13.18	0.32	11.94	0.14
A7	(130, 78)	11.86	0.04	13.26	0.24	11.89	0.11
A8	(172, 72)	11.85	0.05	13.21	0.29	11.92	0.12
A9	(18, 111)	11.94	0.04	13.93	0.43	11.89	0.09
A10	(78, 131)	11.92	0.02	13.22	0.28	12.17	0.37
A11	(134, 128)	11.98	0.08	13.25	0.25	11.97	0.17
A12	(188, 120)	11.87	0.03	13.83	0.33	11.71	0.09
A13	(25, 169)	12.02	0.12	13.85	0.35	11.88	0.08
A14	(77, 184)	11.88	0.02	13.28	0.22	12.16	0.36
A15	(130, 179)	12.10	0.20	13.23	0.27	11.58	0.22
A16	(174, 181)	11.94	0.04	13.25	0.25	11.91	0.11
	Average	11.91	0.01	13.39	0.11	11.92	0.12
